# Vestibular Neurostimulation for Parkinson’s Disease: A Novel Device-Aided Non-Invasive Therapeutic Option

**DOI:** 10.3390/jpm14090933

**Published:** 2024-08-31

**Authors:** K. Ray Chaudhuri, Karolina Poplawska-Domaszewicz, Naomi Limbachiya, Mubasher Qamar, Lucia Batzu, Aleksandra Podlewska, Kristen Ade

**Affiliations:** 1 Basic and Clinical Neuroscience Department, The Maurice Wohl Clinical Neuroscience Institute, Institute of Psychiatry, Psychology and Neuroscience, King’s College London, London WC2R 2LS, UK; naomi.limbachiya@kcl.ac.uk (N.L.); mubasher.qamar@kcl.ac.uk (M.Q.); lucia.batzu@kcl.ac.uk (L.B.); aleksandra.podlewska@kcl.ac.uk (A.P.); 2 Parkinson’s Foundation Centre of Excellence, King’s College Hospital, London SE5 9RS, UK; 3 Department of Neurology, Poznan University of Medical Sciences, 60-355 Poznan, Poland; 4 Scion NeuroStim, Inc., Durham, NC 27707, USA; kade@scionneurostim.com

**Keywords:** Parkinson’s disease, galvanic vestibular stimulation, caloric vestibular stimulation, sensory neuromodulation, non-invasive neuromodulation, vestibular system

## Abstract

Dopaminergic replacement therapy remains the mainstay of symptomatic treatment for Parkinson’s disease (PD), but many unmet needs and gaps remain. Device-based treatments or device-aided non-oral therapies are typically used in the advanced stages of PD, ranging from stereotactic deep brain stimulation to levodopa or apomorphine infusion therapies. But there are concerns associated with these late-stage therapies due to a number of procedural, hardware, or long-term treatment-related side effects of these treatments, and their limited nonmotor benefit in PD. Therefore, there is an urgent unmet need for low-risk adjuvants or standalone therapies which can address the range of burdensome motor and nonmotor symptoms that occur in PD. Recent studies suggest that non-invasive neurostimulation of the vestibular system may be able to address these gaps through the stimulation of the vestibular brainstem sensory network which extensively innervates brain regions, regulating both motor and a range of nonmotor functions. Therapeutic non-invasive vestibular stimulation is a relatively modern concept that may potentially improve a broad range of motor and nonmotor symptoms of PD, even at early stages of the disease. Here, we review previous studies supporting the therapeutic potential of vestibular stimulation for the treatment of PD and discuss ongoing clinical trials and potential areas for future investigations.

## 1. Introduction

The natural history of Parkinson’s disease (PD), while highly variable, often begins with a prodromal period during which a range of nonmotor symptoms such as sleep, mood, and sensory abnormalities present. Over time, the emergence of the cardinal motor features of PD (i.e., bradykinesia, rigidity and/or resting tremor), usually asymmetrical at onset, prompt further clinical evaluation, formal diagnosis, and onset of treatment with oral levodopa therapies, which remain the gold standard in anti-Parkinsonian treatments [1]. Many patients who initiate levodopa treatment initially experience a relatively robust motor symptom response to levodopa, though not all patients experience full motor symptom relief from the therapy and unmet needs remain [2]. Nonmotor symptoms also typically persist during this period as they are not well addressed by dopaminergic therapies and are often undertreated in the early years following diagnosis, largely due to physicians being unaware of them [3]. With time, the responsiveness of motor symptoms to levodopa diminishes, requiring higher dosing for symptomatic relief that often leads to complications such as fluctuations in motor symptoms, levodopa-induced dyskinesias, and worsening of nonmotor neuropsychiatric, autonomic, sleep, and cognitive symptoms [4].

The loss of dopaminergic neurons in the substantia nigra pars compacta leads to abnormal neuronal oscillatory and synchronous activity in the pathways connecting the subthalamic nucleus, globus pallidus pars interna, and cerebral cortex, and is believed to result in key motor symptoms of PD such as tremor, rigidity, bradykinesia, and postural disturbances [5].

When levodopa-induced motor and nonmotor fluctuations appear, device-aided therapies are available for the management of advanced PD (APD). Apomorphine and the recently available foslevodopa/foscarbidopa are subcutaneous infusion therapies while levodopa carbidopa gel and levodopa carbidopa entacapone gel are delivered by an intrajejunal route. The efficacy of these therapies has been shown in pivotal licensing studies and also in long-term studies of apomorphine and levodopa carbidopa gel, which have been in use for over a decade [6].

However, in spite of their proven efficacy, a range of side effects complicate these treatment options; apomorphine infusion is associated with subcutaneous nodules, somnolence, and risk of psychosis such as hallucinations and impulse control disorders. Levodopa carbidopa infusion is associated with device-related complications such as tube dislocation, and tube blockage, in addition to long-term issues such as weight loss and polyneuropathy in some patients [7].

An alternative common approach is through deep brain stimulation (DBS) Ref. DBS applies high-frequency (100–200 Hz) electrical stimulation, usually to the subthalamic nucleus, to mirror the effects of a brain lesion, which alters abnormal signalling and reduces tremors, and improves motor function and dyskinesias severity and frequency [8,9]. Stimulation of other targets within the brain, such as the brainstem nuclei (specifically the pedunculopontine nucleus (PPN)) has been explored but has shown variable results [10]. Although DBS acutely improves motor symptoms and reduces the need for high doses of levodopa, thereby minimising complications, it does not fully address the nonmotor symptoms of PD. The surgical procedure is also risky and can lead to several complications. Therefore, neurosurgical options such as DBS are reserved for PD patients usually below 65–70 yrs age without severe dementia and who are at a low risk of post-surgical complications [8].

Non-invasive brainstem neuromodulation by means of vestibular stimulation is an emerging investigational approach that may be able to address several of the limitations of current standard of care therapies for PD. In this narrative review, we discuss the available evidence and rationale for this exciting development, specifically in relation to Parkinson’s disease (PD) and nonmotor symptoms which are an important continuing unmet need in relation to management of the disease [2].

## 2. The Vestibular System

**Figure 1:**
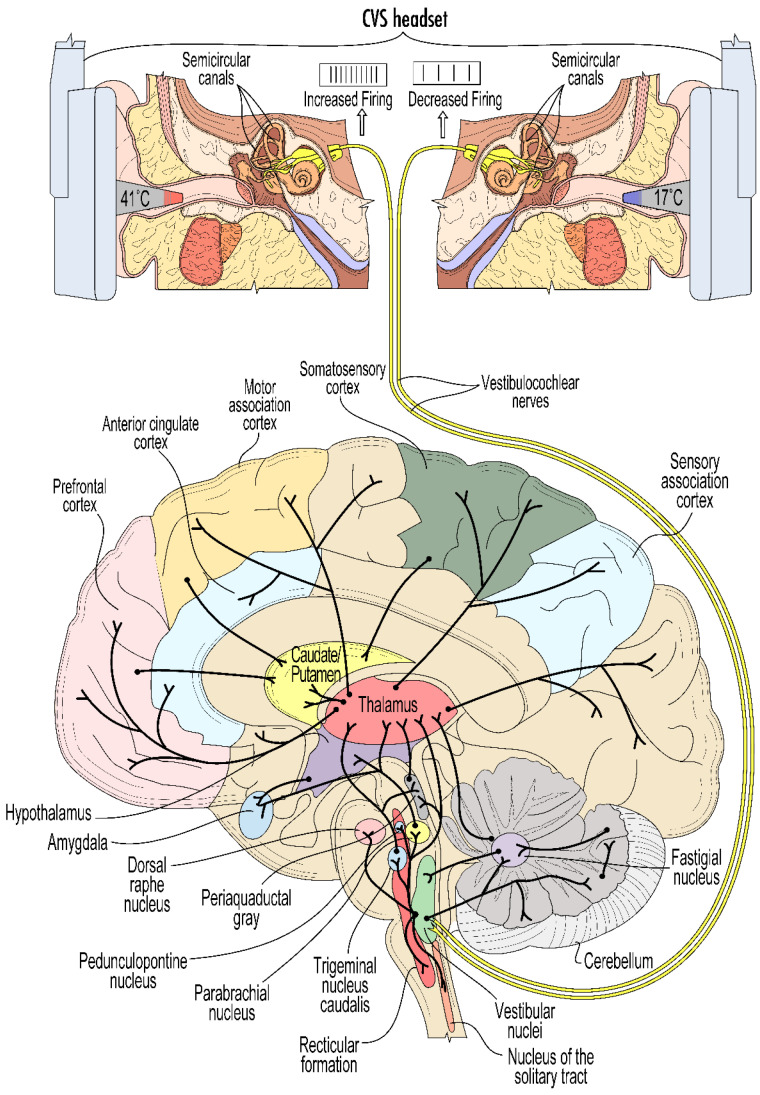
A schematic view showing the mechanism of action of CVS and projections of the vestibular nuclei to brain regions implicated in PD and/or migraine. The action potential firing rates of the vestibulochoclear nerves are increased by the warming of the left ear and decreased by cooling of the right ear by means of the tvCVS solid-state device. These nerves innervate the vestibular nuclei in the brainstem that send both direct and indirect excitatory afferents to a number of brainstem nuclei implicated in migraine pathology. The vestibular nuclei also show extensive polysynaptic connectivity with numerous structures in the midbrain and forebrain affected in PD and migraine pathology. The orientation of the brain has been turned 90° to show connectivity in a sagittal cross-section. Modified figure from (Black and Rogers 2020 [14]).

The vestibular system (VS) is a sensory network made up of both peripheral and central nervous system components. The vs originates in the inner ear and reaches throughout the brainstem and higher brain regions. Three semi-circular ducts that are oriented at right angles to each other are found in each ear. Fluid within these canals moves with head movements and pushes on hair cells, which translate rotational movement in any plane to electrical signals. These signals are sent to brainstem vestibular nuclei via the vestibulocochlear nerve. The vs is responsible for the sensory processing of head motion that is critical to maintaining balance and coordinating movement [11]. Motion-induced stimulation of the vs initially involves the fluid within the vestibular apparatus in the inner ear, which is comprised of three orthogonally positioned semi-circular canals and two otolith structures (the utricle and the saccule). When the head moves, the resultant motion of the fluid within these structures leads to the displacement of mechanoreceptors called hair cells positioned within the cupula and otolithic organs that translate mechanical movements into electrical signals. The deflection of the hair cells mechanically opens or closes potassium channels, depending on the direction of fluid motion, and the resulting depolarisation or hyperpolarisation increases or decreases synaptic transmission, thereby affecting the tonic firing rate of the vestibular afferents. For a comprehensive review of the structures and pathways of the vestibular system, see (Khan and Chang 2013) [11]. The changes in the firing rate of the vestibular afferents subsequently modulate the activity of neurons in the brainstem vestibular nuclei, and via synaptic and polysynaptic relays from those nuclei, modulate a multitude of connected regions in the brainstem, cerebellum, thalamus, and cortex via extensive upward and lateral projections [12] (Figure 1).

In addition to motion-induced modulation, the activity of vestibular afferents can also be modulated using non-invasive techniques by means of galvanic vestibular stimulation (GVS), or caloric vestibular stimulation (CVS) [13]. These methods are further described in the following sections.

## 3. The Vestibular System and Parkinson’s Disease

Previous tracing and functional connectivity studies have provided evidence to support monosynaptic or polysynaptic connectivity of the vestibular nuclei to brain regions implicated in the motor symptoms of PD including the dorsolateral striatum (caudate/putamen) [15,16,17].

The projections from the vestibular nuclei to the dorsolateral striatum appear to be involved with expression of the following:

- Bradykinesia [18];
- Gait [19] and possibly postural stability [20] (via the PPN and the cerebellum [21,22]);
- Resting tremor and levodopa-induced dyskinesias [23,24].

Moreover, the vestibular nuclei have direct and indirect inputs into brain regions implicated in the nonmotor symptoms (NMS) of PD, including the (1) corticolimbic network (consisting of the anterior cingulate cortex, dorsolateral prefrontal cortex, amygdala, and hippocampus), the dorsal raphe nucleus (DRN), and the parabrachial nucleus implicated in depression and anxiety [22,25]; (2) the sensory association cortices, temporal–parietal regions, prefrontal cortex, locus coeruleus (LC), peri-sylvia, PPN, cerebellum, and hippocampal structures [26] implicated in memory deficits and cognitive impairment; (3) the periaqueductal grey (PAG) [27] (Ma, Li et al., 2023) implicated in blood pressure and orthostatic hypotension [28] and bladder control, the latter of which is also regulated by the hypothalamus, cerebellum, basal ganglia, and frontal cortex [29]; and (4) the PPN and DRN implicated in sleep, arousal, and visual hallucinations [30]. Also of note, the vestibular nuclei have direct neural inputs to the thalamus, which regulates the functional connectivity of multiple cortical regions, contributes to most aspects of brain function, and has been implicated in several nonmotor symptoms of PD [31]. Additionally, the connectivity with the serotonergic raphe nuclei and the noradrenergic LC, the main structures affected by the neurodegenerative processes in PD and thought to be involved in the recently described noradrenergic subtype of PD and both noradrenaline (NA) and 5-hydroxytryptamine (5-HT), are involved in the declining pain modulatory pathway functionality [32]. Additionally, vestibular connectivity with the parabrachial nucleus stimulation suggests the possibility for vestibular stimulation to modify the autonomic, affective, and emotional aspects of pain [12].

Further support for the vestibular regulation of motor and nonmotor symptoms in PD can be derived from observations of comorbidities associated with vestibular dysfunction. For instance, one study investigating patients with chronic neurotological dysfunction, such as vestibular migraine, reported concurrent symptoms of anxiety, depression, fatigue, and daytime sleepiness [33]. Additionally, a U.S. survey of patients with vestibular vertigo highlighted significant cognitive and neuropsychiatric issues [34]. Recent evidence also suggests that vestibular dysfunction may contribute to motor-related deficits in PD, including postural instability, gait dysfunction motor phenotype, and freezing of gait [35].

## 4. Methods for Vestibular Stimulation

### 4.1. Galvanic Vestibular Stimulation

GVS is delivered through the application of low amplitude (<−2 mA) transcutaneous electrical current, applied to electrodes placed on the bilateral mastoid processes to stimulate vestibular hair cells and irregularly firing vestibular afferents [13]. There are several possible electrode configurations that can be used for GVS, including bilateral monopolar, unilateral monopolar, or bilateral bipolar configurations, though the latter is most common in therapeutic investigational applications [36]. The current applied can be a direct current, alternating current, or stochiastic noisy waveforms [37]. GVS works by creating a voltage bias between the two sets of vestibular organs. It is generally agreed that GVS exerts stronger effects on the subset of vestibular afferent neurons that fire irregularly, though the specific sites of action are still debated within the literature. While GVS was originally proposed to primarily act on either the otoliths or on the semicircular canals of the inner ear [38], more recent work indicates that both structures are likely involved [13,39].

### 4.2. Caloric Vestibular Stimulation

CVS is an established technique that utilises thermal energy to modulate activity of the vestibular afferents. CVS is commonly used to clinically diagnose disorders of balance and evaluate brainstem function in the assessment of brain death [40]. The process traditionally uses water or air irrigators to warm or cool the external auditory canal of patients, changing the density of the endolymphatic fluid in the semicircular canals. The density change induces flow of the endolymph, modulating the firing rate of vestibular hair cells, and consequently modulating the activity of the vestibular afferents. CVS primarily affects the horizontal semicircular canal, which protrudes into the middle ear space, and thereby, is the first structure to intersect a thermal wave formed by CVS. However, there is evidence that the other canals, as well as the utricle, also respond to caloric stimulation [41,42]. There is some debate about the path that heat takes from the external ear canal to the inner ear, but the preponderance of evidence confirms that conduction along the petrous bone (part of the temporal bone) dominates [40]. Warming temperatures increase while cooling temperatures decrease the tonic firing rate of the vestibulocochlear nerves which project to the vestibular nuclei in the brainstem.

**Figure 2:**
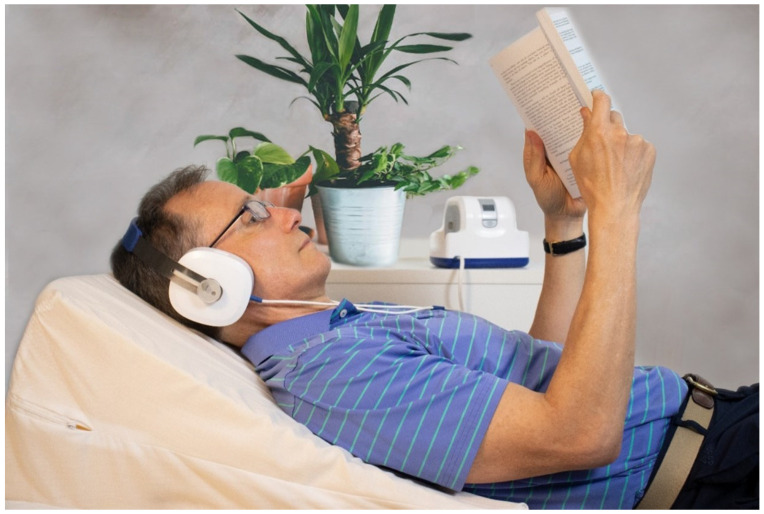
Time-varying caloric vestibular stimulation (tvCVS) treatment delivery. Image shows a participant lying on an incline wedge pillow while undergoing treatment with a solid-state tvCVS headset device.

Despite the long-standing history of safe use in the diagnostic setting, the therapeutic potential of traditional water or air irrigation CVS methods has been hampered by challenges associated with modulating temperatures specifically or rapidly, difficulties associated with controlling dosing, and adaptation following constant-temperature CVS. Recent advancements in CVS technology have led to the development of solid-state devices capable of delivering time-varying CVS (tvCVS) through small metal earpieces that sit inside the ear canals. The solid-state tvCVS devices allow for a continuous change in temperature for the sustained modulation of vestibulocochlear nerve activity and avoidance of the ciliary adaptation that occurs within minutes of constant-temperature irrigation CVS [14]. Furthermore, the solid-state device facilitates a controlled and slowed time-rate-of-change in the thermal stimulus, which could limit the common side effects associated with irrigation-based CVS, such as dizziness, nausea, and vomiting. Notably, the portable, headset-like design of the solid-state tvCVS device allows for at-home application of tvCVS therapy (Figure 2). Although early human studies with tvCVS devices have demonstrated promising outcomes for both motor symptoms and NMS of PD [43,44]. these benefits have yet to be evaluated in rigorous, large-scale clinical trials.

### 4.3. Broad Physiological Effects of Vestibular Stimulation

Both GVS and CVS can modulate broad activity throughout the brain via endogenous networks of several brainstem nuclei that have projections to the midbrain, thalamus, and cortex [45]. This broad neuromodulation from vestibular stimulation has been evidenced through several neuroimaging studies [14,46,47]. The means to modulate activity across broad regions of the brain separates vestibular stimulation from other conventional forms of non-invasive neurostimulation like transcranial magnetic stimulation (TMS) and transcranial direct or alternating current stimulation (tDCS and tACS, respectively), which offer a top-down approach and stimulate a focused and narrow target, primarily limited to the neocortex [48]. Furthermore, because GVS and CVS act through a sensory system, the applied stimulus is modified by the sensory organ in a way that is matched to the native neuronal dynamics [14,38,45], whereas with other forms of non-invasive neurostimulation including TMS, tDCS, and tACS, there is a mismatch between the applied tACS signal and endogenous neuronal network frequencies [36].

Vestibular stimulation may also involve the release of several neurotransmitters including serotonin, histamine, acetylcholine and gamma aminobutyric acid (GABA) [27,49,50,51]. These neurotransmitters are closely involved in the pathophysiology of cognition and neuropsychiatric dysfunction, as well as a number of other nonmotor symptoms (NMS) of Parkinson’s disease (PD), nonmotor subtypes, and stepped care for PD [52,53,54,55].

Several studies have demonstrated the broad physiological effects of vestibular stimulation related to neurological processes throughout the brain [56,57,58]. The use of GVS in rat models has been shown to induce hippocampal cell proliferation as well as striatal reduction in c-Fos expression and the release of serine and threonine [56,57,58]. In humans, GVS treatment has been shown to effect changes in EEG patterns such as power spectra and P300 morphology as well as balance and postural sway [38,59]. Previous reports have indicated tvCVS alters blood flow and induces oscillations in measures of cerebrovascular resistance [40]. It has been hypothesised that the changes in cerebral blood flow that result from coordinated tvCVS neurostimulation may enhance neuronal and cerebrovascular integrity. However, these hypotheses have not yet been tested experimentally, and the mechanism(s) of action has yet to be determined. Functional magnetic resonance imaging (fMRI) studies also indicate that tvCVS modulates activity in several brain regions implicated in memory and cognition, including the hippocampus, cerebellum, operculum, and frontal and parietal lobules, following both irrigation-based CVS and tvCVS [14], and therefore, may be of relevance to cognitive decline and dementia in PD [46,60,61,62]. Additionally, a pivotal trial has demonstrated that tvCVS significantly reduced the number of migraine days in individuals suffering from episodic migraine pain [63], while smaller studies have provided evidence that irrigation-based CVS may have pain-relieving effects in multiple pain conditions including central post-stroke pain, phantom limb pain, and persistent pain following myelitis of the cervical spinal cord [64,65,66].

### 4.4. Galvanic Vestibular Stimulation and Parkinson’s Disease

GVS has been reported to provide a therapeutic benefit for motor aspects of PD in some animal studies and human case studies. Samoudi et al. demonstrated that noisy GVS both improved rotarod performance and increased GABA release in the substantia nigra of 6-hydroxydopamine hemi-lesioned parkinsonian rats [67]. In small human laboratory-based studies, single sessions of direct current GVS has been to be associated with reduction in postural instability [68] as well as improvements in finger tapping speed and the Timed Up and Go test [69]. In another study, Pal et al. examined anterior-posterior and side-to-side sway in PD and reported a minor benefit after subsensory, noisy GVS [70]. A later study evaluating responses to the “pull test” in 10 PD subjects demonstrated that subsensory, noisy GVS was associated with shorter postural corrections and quicker regain of posture when pulled back unexpectedly [51]. Case studies also suggest improved visuo-motor tracking and rapid rates of rest-to-active motor transitions with GVS [71]. GVS treatments have been shown to normalise the functional connectivity of the PPN, the pallidum, the inferior parietal cortex, and the cerebellar cortices [72]. These observations in motor-related neural pathways suggest that GVS treatment may lead to improvements in motor behaviour outcomes for neurodegenerative conditions such as PD. However, to date, GVS has only been delivered in clinical laboratory settings, thus limiting the translatability of GVS as a viable longitudinal therapy. Additionally, significant work is required to further understand the variability in response and the impact of the wide range of stimulation parameters afforded by GCS to support future clinical adoption of this emerging technology [71].

### 4.5. Caloric Vestibular Stimulation and Parkinson’s Disease

The bulk of the clinical data related to vestibular stimulation as a potential treatment for PD relates to tvCVS. The first evidence to support the potential efficacy of tvCVS to treat motor and nonmotor symptoms of PD came from a case study reporting that a single participant showed an approximate 50% reduction in both motor symptoms and NMS associated with PD following 8 weeks of twice daily tvCVS treatments, self-administered in a home setting with a solid-state device [43].

The investigators followed up on this finding by performing a single-site, randomised controlled trial in which 46 people diagnosed with PD and taking stable doses of medications self-administered tvCVS, twice daily over a period of 8 weeks. Participants in the active tvCVS group demonstrated reduced ON-state (levodopa-medicated) motor signs, based upon the International Parkinson’s and Movement Disorder Society-Unified Parkinson’s Disease Rating Scale part III (95% CI for therapeutic gains between the active and control group: −13.2 to −1.8 points). Additionally, NMS burden according to the Non Motor Symptom Scale was reduced (95% CI for therapeutic gains between the active and control group: −45.9 to −13.5 points). This reduction in nonmotor symptoms was driven by reductions across a broad spectrum of NMS domains including sleep/fatigue, mood/cognition, attention/memory, gastrointestinal function, urinary function, sexual function, and miscellaneous NMS [43,44].

Notably, these clinical benefits in both motor and nonmotor domains showed evidence of persistence one-month post-treatment. Based on these observations, a large-scale, multicentre, pivotal randomised controlled trial with an open label extension to evaluate the safety and efficacy of tvCVS for the treatment of PD symptoms, called the STEM-PD (NCT04797611, NCT04799418), is currently under way. Other ongoing, early-stage clinical trials in people with Parkinson’s are further exploring the potential of tvCVS treatments for postural instability and gait difficulties (NCT04768647) and dementia (NCT05987540). While the pivotal trial (NCT04797611) is evaluating tvCVS as an adjuvant treatment for a broad spectrum on NMS in PD, given the connectivity of the vestibular system to key regions implicated in the NMS pathology, more focused studies may also be warranted to further evaluate the potential of tvCVS to treat specific NMS, including memory and cognitive dysfunction, neuropsychiatric problems such as depression and anxiety, Parkinson’s pain, and autonomic dysfunction such as orthostatic hypotension and urinary incontinence.

## 5. Conclusions

Modern management of PD is complicated, as it needs to encompass a range of motor and nonmotor symptoms, address subtype-specific personalised therapies, as well as incorporate appropriate patterns of advanced therapies for advanced PD. Several non-oral invasive therapeutic options ranging from apomorphine and levodopa subcutaneous infusion to intrajejunal levodopa delivery, as well as refined methods of DBS, exist and provide effective symptom control. But these therapies are all associated with a degree of intolerance in the long run due to device-related or treatment-related side effect issues. Poor management of NMS of PD also remains a continuing challenge. Non-invasive therapies that can effectively manage the motor symptoms of PD while also addressing key nonmotor symptoms of PD are therefore a highly desirable option.

The benefits of vestibular stimulation for treating PD may be able to plug this gap in care. The preliminary evidence supporting the use of vestibular stimulation to treat both motor and nonmotor symptoms of PD is compelling. However, it is important to note that the majority of this evidence has relied on pre-clinical animal studies, and vestibular stimulation devices designed for human use remain limited to investigational use for PD indications. Although it is too early to draw any specific clinical conclusions, data from ongoing trials such as the STEM-PD (NCT04797611, NCT04799418) may provide a clearer picture of the benefits provided from such interventions. Future implications remain speculative but the results of the ongoing STEM-PD pivotal trial will elucidate whether tvCVS may serve as safe and effective adjuvant or alternative to standard of care oral dopaminergic therapies for treating both nonmotor symptoms and motor symptoms in Parkinson’s. Future clinical trials would be required to establish whether tvCVS may be beneficial in drug-naïve de novo PD as well as specific nonmotor subtypes of PD.

## Data Availability

Not applicable.
